# Sleep Apnea: The Slept-Upon Cardiovascular Risk Factor

**DOI:** 10.3390/biomedicines13102529

**Published:** 2025-10-16

**Authors:** Adriana-Loredana Pintilie, Dragos Traian Marius Marcu, Andreea Zabara-Antal, Raluca-Ioana Arcana, Diana-Gabriela Iosep, Mihnea Miron, Carina-Adina Afloarei, Mihai-Lucian Zabara, Radu Crisan Dabija

**Affiliations:** 1 Clinical Hospital of Pulmonary Diseases Iași, 700116 Iași, Romania; pintilie.adriana.loredana.mg6.7@gmail.com (A.-L.P.); andreeazabara@yahoo.com (A.Z.-A.); raluca.dospinescu@yahoo.com (R.-I.A.); carina_adina98@yahoo.com (C.-A.A.); radu.dabija@umfiasi.ro (R.C.D.); 2 Grigore T. Popa University of Medicine and Pharmacy, 700116 Iași, Romania; diana.iosep@umfiasi.ro (D.-G.I.); miron_mihnea@d.umfiasi.ro (M.M.); mihai-lucian.zabara@umfiasi.ro (M.-L.Z.); 3 Department of General Surgery and Liver Transplantation, St. Spiridon University Hospital Iași, 700115 Iași, Romania

**Keywords:** cardiovascular involvement, sleep apnea, CPAP

## Abstract

**Background:** Obstructive sleep apnea (OSA) is prevalent and often underdiagnosed in cardiology. Worldwide, approximately 936 million adults aged 30–69 are affected by OSA, with the highest numbers in the USA, China, Brazil, and India. In cardiovascular clinics, OSA is found in about 40–80% of patients with hypertension, heart failure, coronary artery disease, atrial fibrillation, or stroke. Meta-analyses link OSA to nearly twice the risk of cardiovascular disease, stroke, and all-cause mortality. Continuous positive airway pressure (CPAP) therapy addresses the underlying mechanisms of OSA and enhances intermediate cardiovascular indicators. **Materials and Methods:** We conducted a narrative review using major medical search engines (PubMed, Embase, Cochrane) to examine recent statements, meta-analyses, large cohorts, and key trials. The review focused on the cardiovascular burden of sleep apnea and its pathophysiology—including arrhythmic, hemodynamic, vascular, and coagulation aspects—as well as the effects of CPAP on intermediate cardiovascular outcomes. We aimed to provide a synthesised overview of current cardiovascular evidence related to the burden and mechanisms of OSA, and to summarise the effects of continuous positive airway pressure (CPAP) on intermediate and clinical cardiovascular outcomes. **Results:** Intermittent hypoxia, sleep fragmentation, and major negative fluctuations in intrathoracic pressure create a clear pathway leading to adverse cardiovascular outcomes. This occurs through mechanisms like sympathetic activation, RAAS activation, endothelial dysfunction, oxidative stress, and inflammation, linking OSA to these health issues. Studies show that greater severity of OSA correlates with higher cardiovascular risk, including increased incidence and recurrence of AF, resistant hypertension, and new cases of heart failure. CPAP effectively lowers AHI and enhances nocturnal oxygen levels, as well as intermediate cardiovascular indicators such as blood pressure, sympathetic activity, and certain aspects of ventricular function, with clinical benefits most evident in adherent patients. **Conclusions:** OSA is a significant, modifiable risk factor for cardiovascular disease. Routine cardiovascular care should include targeted screening for OSA, especially in cases of resistant hypertension, atrial fibrillation, and heart failure, along with timely sleep testing and adherence-focused CPAP therapy, in addition to traditional risk-reduction methods.

## 1. Introduction

Obstructive sleep apnoea (OSA) is a prevalent form of sleep-related breathing disorder, with a notably high incidence among patients with cardiovascular disease [1]. Globally, approximately 936 million adults aged 30–69 are affected by OSA, with mild cases, while about 425 million have moderate forms. The highest numbers are in the USA, China, Brazil, and India. Conversely, lower estimates are reported in Hong Kong, Macao, and New Zealand [2]. OSA consistently correlates with hypertension, stroke, coronary artery disease (CAD), atrial fibrillation, and heart failure across regions. Asian cohorts tend to have nocturnal hypertension or non-dipping blood pressure at lower body mass indexes, and in the USA, strokes, heart failure, atrial fibrillation, and atherosclerosis occur more frequently [3,4]. Adult Obstructive Sleep Apnea (OSA) is defined in accordance with the standards established by the American Academy of Sleep Medicine (AASM), utilizing data acquired from polysomnography or home sleep testing using polygraphic devices. It categorises obstructive apneas based on severity, which is determined by the number of events per hour of sleep, referred to as the Apnea-Hypopnea Index (AHI). The classifications include mild (AHI 5–15 per hour), moderate (AHI 15–30 per hour), and severe (AHI ≥ 30 per hour). An obstructive apnea is recorded when airflow decreases by ≥90% from baseline for at least 10 s, accompanied by continued respiratory effort. A hypopnea is characterised by a reduction in airflow of ≥30% for at least 10 s, associated with either a ≥3% oxygen desaturation and/or an EEG change in arousal.

OSA’s prevalence is recorded at as high as 40–80% among patients with hypertension, heart failure, coronary artery disease, pulmonary hypertension, atrial fibrillation, and stroke [5]. Yet, it remains under-recognised and undertreated within routine cardiology care. A meta-analysis involving 25,760 individuals demonstrated a nearly doubled risk of cardiovascular disease, stroke, and all-cause mortality, with a 17% increase in cardiovascular risk associated with every 10-unit increase in the number of events [6]. Furthermore, women were found to have a 28% higher risk. Patients with OSA had an 8% greater risk of major atherosclerotic cardiovascular adverse events, such as myocardial infarction, stroke, unstable angina, or heart failure, compared to individuals without OSA after a five-year follow-up [7]. In a Danish cohort comprising over 20,000 individuals under the age of 50 with OSA, the five-year risk of any cardiovascular event was nearly doubled relative to matched controls, with a particularly elevated risk for incident hypertension [8]. It is noteworthy that, at the five-year follow-up, 27.3% of patients with OSA developed incident hypertension, in contrast to 15.0% of healthy individuals. Regarding resistant hypertension, multiple studies have indicated that individuals with OSA and risk factors for both sleep apnoea and cardiovascular involvement exhibit a higher risk of resistant hypertension compared to non-OSA individuals [9]. The association with OSA significantly increases the risk of atrial fibrillation, with an odds ratio of 1.71 for new-onset fibrillation, 2.65 for postoperative arrhythmia, and 2.93 for AF recurrence after ablation, which increases proportionally with the severity of sleep apnea [10]. Heart failure shows an increase in individuals diagnosed with severe OSA, based on the data provided by the Swedish SAPIS prospective cohort. Thus, severe OSA independently predicted incident heart failure with an adjusted hazard ratio of 2.42, after a median follow-up of 8.8 years [11]. Regarding pulmonary hypertension, literature data suggest that the pooled prevalence of pulmonary hypertension among OSA patients was 36%. The co-occurrence of both OSA and pulmonary hypertension was associated with more severe respiratory impairment, male sex, older age, higher body mass index and a higher number of respiratory events [12].

Obesity is the most significant modifiable risk factor for OSA, with risk increasing notably across various BMI levels. A study involving 12,860 participants revealed that overweight individuals are about twice as likely to develop OSA, while obese individuals face roughly five times the risk compared to those with a BMI below 25 kg/m^2^ [13]. Additionally, each 1 kg/m^2^ rise in BMI correlates with a 14% increase in AHI and a 61% higher probability of moderate to severe OSA [14]. Long-term data indicate that a 10% weight gain can raise AHI by 32% and increase the likelihood of developing moderate to severe sleep-disordered breathing sixfold [15]. Despite the strong association with obesity, over 20% of OSA cases occur in non-obese individuals [16]. The GLP-1 receptor and dual GIP/GLP agonists can lower weight and improve obesity-related OSA. In the SCALE sleep apnea trial, 3.0 mg of liraglutide administered over 32 weeks reduced AHI by 12.2 events per hour and resulted in an average weight loss of 5.7%. Weekly tirzepatide at 10/15 mg doses in adults with moderate to severe OSA and obesity decreased AHI by 20 events per hour and achieved 18–20% weight loss, leading to FDA approval of tirzepatide for treating OSA in obese adults in 2024 [17]. Genetic evidence from instrumental variables indicates that OSA causally contributes to atrial fibrillation (AF), beyond obesity. Genome-wide association studies reveal that a genetic predisposition to OSA correlates with an increased risk of AF [18]. Additionally, mediation models estimate that around 22% of the link between body mass index and AF is mediated by OSA, while 49% is mediated by circulating leptin. Collectively, these mediators explain roughly 88% of the excess risk [19].

The pathophysiological effects of OSA are intricate and involve multiple factors, with mechanisms that are not fully understood. Repeated episodes of upper airway collapse lead to several key consequences: intermittent hypoxia, disrupted sleep, negative intrathoracic pressure, endothelial dysfunction, activation of the sympathetic nervous system and the renin–angiotensin–aldosterone system, and increased oxidative stress. These factors contribute to endothelial damage, inflammation, and nocturnal blood pressure spikes. Together, they promote nocturnal hypertension, create an arrhythmogenic substrate, and put pressure-volume load on both ventricles, eventually causing heart failure. Hypertension, atrial fibrillation, and heart failure, in turn, worsen OSA, forming a bidirectional cycle. Due to this potential negative feedback loop, managing cardiovascular comorbidities associated with OSA remains essential.

The first-line therapy for moderate to severe obstructive sleep apnea (OSA) is continuous positive airway pressure (CPAP). This intervention eliminates obstructive apneas or hypopneas, enhances nocturnal oxygenation and hemodynamic stability, and improves sleep quality, neurocognitive function, mood, and overall quality of life.

Our goal was to compile current cardiovascular research on obstructive sleep apnoea to determine its prevalence in the general and cardiology populations. We aimed to identify key underlying mechanisms such as intermittent hypoxia, fluctuations in intrathoracic pressure, activation of the sympathetic nervous system and RAAS, endothelial dysfunction, and thrombogenicity. Additionally, we evaluated how CPAP therapy influences both intermediate and major cardiovascular outcomes. This review provides clinicians with a practical framework to recognise and assess the most relevant cardiovascular outcomes associated with OSA and the expected effects of CPAP.

**Figure 1:**
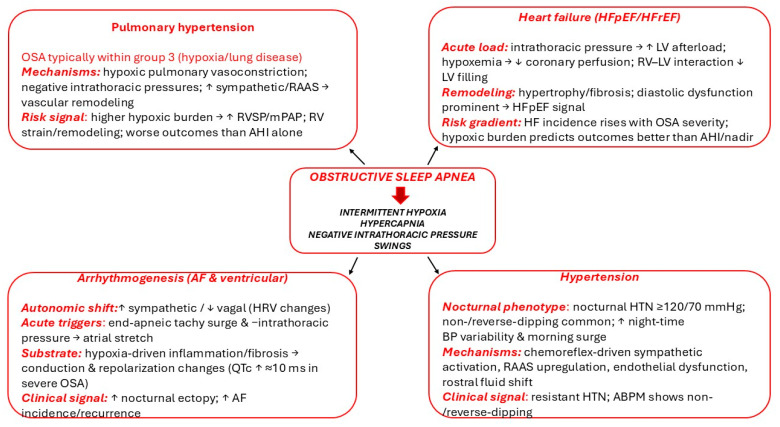
OSA-related intermittent hypoxia, hypercapnia, large negative intrathoracic pressure swings and sleep fragmentation trigger sympathetic and RAAS activation, leading to endothelial dysfunction, inflammation, oxidative stress, and nocturnal blood-pressure surges. ↓: decrease, ↑: increase.

Figure 1 shows the pathophysiological cascade whereby OSA-related intermittent hypoxia, hypercapnia, large negative intrathoracic pressure swings, and sleep fragmentation activate the sympathetic nervous system and RAAS, leading to endothelial dysfunction, inflammation, oxidative stress, and nocturnal blood pressure surges. 

## 2. Materials and Methods

We conducted a thorough literature review using the databases PubMed, Google Scholar, Embase, and Cochrane to identify key studies, large cohort datasets, and recent systematic meta-analyses related to the cardiovascular implications of sleep apnoea published from 2015 to 2025.

A total of 432 records were identified; 121 full-text articles were screened, and 69 studies met the inclusion criteria: peer-reviewed, English-language human studies involving adults with obstructive sleep apnea that evaluated cardiovascular consequences and/or the impact of positive airway pressure. Eligible endpoints encompassed hypertension phenotypes (including nocturnal, non-dipping, and reverse dipping), atrial fibrillation and other arrhythmias, heart failure (HFpEF/HFrEF), coronary artery disease, endothelial function and arterial stiffness (such as flow-mediated dilation and pulse wave velocity), carotid intima–media thickness, coagulation and hemostasis markers, and pulmonary hypertension, as well as the effects of CPAP on these outcomes. The inclusion criteria comprised randomized trials, cohort studies, case–control studies, cross-sectional studies, and high-quality systematic reviews and meta-analyses. Conversely, we excluded case reports, conference abstracts, animal or pediatric studies, and articles lacking measurable cardiovascular endpoints or a clear association between OSA and cardiovascular conditions. Two reviewers independently screened titles/abstracts and full texts, with disagreements resolved by consensus. Where possible, we prioritised higher-level evidence (trials, meta-analyses) and clinically meaningful outcomes. Mechanistic and intermediate endpoints (e.g., endothelial function, arterial stiffness, coagulation markers) were summarised to contextualise the pathways.

We constructed database-specific search strings combining controlled vocabulary and keywords for obstructive sleep apnea (OSA/OSAS/SDB) and cardiovascular outcomes, including hypertension phenotypes (resistant, nocturnal, non-/reverse-dipping), arrhythmias (atrial fibrillation, ventricular ectopy, HRV), heart failure (HFpEF/HFrEF, diastolic dysfunction), coronary/vascular disease (CAD, MI, ACS, endothelial dysfunction, FMD, PWV, CIMT), hemostasis (hypercoagulability, platelet activation, fibrinogen, vWF, PAI-1), pulmonary hypertension, mechanistic intermediates (intermittent hypoxia, hypoxic burden, oxidative stress, RAAS/aldosterone), and interventions (CPAP/APAP/PAP, BiPAP, mandibular advancement devices). Given the heterogeneity in populations, OSA definitions, and outcomes, findings were synthesised narratively without meta-analysis; when multiple studies overlapped in cohorts, the most comprehensive and least biased report was preferentially cited. We summarised the key characteristics and principal findings of included studies in Appendix A Table A1. The table lists each study’s year, design, population, aim, principal results, and outcome domain.

## 3. Blood Pressure in OSA: Pathophysiology, Nocturnal Patterns, and Treatment Effects

Blood pressure naturally follows a circadian rhythm, dipping approximately 10–20% at night compared to daytime levels, often referred to as the “dipper” profile. Upon waking, it quickly rises in the morning, a phenomenon known as the morning blood pressure surge (MBPS). This surge results from the activation of the sympathetic nervous system and the hypothalamic–pituitary–adrenal axis, which releases catecholamines and cortisol as the body transitions from sleep to wakefulness. In the hours after waking, blood pressure generally stabilises into a relatively steady phase called the daytime plateau, regulated by both circadian rhythms and daily activities [20]. Importantly, the dipper profile is considered protective for the heart; research shows it is linked to a significantly lower risk of major cardiovascular events compared to other abnormal blood pressure patterns [21].

The episodes of hypoxia and hypercapnia serve as chemoreceptor–mediated stimuli, triggering the efferent sympathetic nerves and the neuronal release of norepinephrine. Secondary to episodes of apnea or hypopnea, there are intermittent surges in sympathetic nervous system activity, causing an elevation in blood pressure and heart rate. After an apneic event, increased breathing stimulates discharge in the stretch afferents of the lungs, which, in turn, inhibits cardiac vagal parasympathetic activity, resulting in higher blood pressure and heart rate. This activation of the sympathetic nervous system persists during the daytime, making OSA a significant contributor to diurnal hypertension [22]. During hypoxic peaks, systolic blood pressure rises by approximately 25 mmHg above the mean values, with about 50% of nocturnal hypertensive patients exceeding 160 mmHg [23].

When nocturnal respiratory events are absent, blood pressure typically decreases during the night. Ambulatory Blood Pressure Monitoring (ABPM) devices are capable of measuring nocturnal blood pressure levels. Nocturnal hypertension is characterised by readings of ≥120/70 mmHg, and OSA can modify these typical values, leading to different nocturnal patterns.

A non-dipping nocturnal pattern, where the reduction in blood pressure is less than 10%, indicates moderate to severe obstructive sleep apnea (OSA), affecting approximately 84% of individuals diagnosed with the condition. This pattern is commonly associated with nocturnal hypertension, characterised by an exaggerated morning surge and increased short-term variability during nighttime [21].

Among blood pressure patterns, the riser or reverse dipper, characterised by a lack of the typical decline or a rise in blood pressure, is most strongly linked to higher risks of cardiovascular events such as stroke, myocardial infarction, and cardiovascular death. The occurrence of OSA was 73.5% when the reverse systolic dipper pattern was seen, exceeding rates in normal, extreme, and reduced dippers. Reverse systolic dipping was identified as an independent predictor of OSA, with reduced and reverse diastolic dipping increasing the odds by about 2.7 and 3.5 times, respectively [24].

Extreme dipping, characterised by a nocturnal blood pressure drop of ≥20%, is not the usual blood pressure phenotype associated with OSA. Its prevalence among OSA patients was 46.2% in systolic categories and 33.3% in diastolic categories, making this pattern less common than reduced and reverse dipping patterns [25].

In hypertensive OSA individuals, 12-week CPAP showed effects that depended on the circadian pattern. Non-dippers experienced significant reductions, with an average nighttime decrease of 4.4 mmHg, while non-dippers showed non-significant changes [20]. The treatment by pattern interaction favoured non-dippers, with a mean reduction of 2.99 mmHg, indicating that CPAP mainly lowers nocturnal BP and partially restores dipping in non-dipper OSA. Among normotensive men with OSA, nocturnal blood pressure increased and their dipping status decreased as severity worsened (night SBP 119 mmHg, with a mean dip of 11%). ODI independently predicted non-dipping status. Using CPAP for over 18 months was associated with lower night-time systolic blood pressure, a larger average dip, and an improvement in dipping status from 25% to 45%, indicating a partial restoration of the dipping pattern [26]. In the HIPARCO randomised trial, individuals with resistant hypertension who were diagnosed with severe OSA experienced, after 12 weeks of CPAP therapy and 24 h monitoring, a mean reduction of 3.1 mmHg and an increase in the proportion of conversions to dipping status by 35.9% [24]. After 8 weeks in untreated hypertensive OSA, valsartan 160 mg reduced 24 h mean BP by 9.1 ± 7.2 mmHg on ABPM, whereas CPAP caused a more modest 2.1 ± 4.9 mmHg reduction; the BP decrease with CPAP showed a dose–response to nightly use (mean adherence 4.8 ± 2.1 h per night) [27].

Emerging data suggest a bidirectional OSA-aldosterone loop, where promoting fluid retention leads to peripherally mediated oedema and worsens OSA by mediating aldosterone production. Meanwhile, OSA-related intermittent hypoxia and chemoreflex activation increase the renin-aldosterone output. These theories are supported by the fact that individuals diagnosed with OSA exhibit upregulated RAAS activity, characterised by higher plasma renin activity, aldosterone, and angiotensin II, alongside hemodynamic overload, as indicated by elevated systolic and diastolic blood pressures and heart rate. Hyperaldosteronism influences OSA due to aldosterone-induced fluid accumulation, causing a rostral fluid shift and parapharyngeal oedema. Coupled with neck tissue congestion, this increases upper airway resistance, leading to collapse and worsening OSA [28]. Data from studies suggest that eplerenone use along with other antihypertensive agents reduces AHI, neck circumference, and blood pressure, as well as aortic pulse wave and arterial wall stiffness [29].

In otherwise healthy, normotensive patients with moderate to severe OSA (no comorbidities), one month of CPAP therapy decreased plasma aldosterone levels from 149 ± 18 pmol/L before CPAP to 109 ± 10 pmol/L after CPAP, with a significant decline in mean BP. It is important to note that in men, aldosterone levels decreased significantly from 198 to 114 pmol/L, and norepinephrine levels decreased in both sexes [30]. When discussing resistant hypertension, the addition of spironolactone to the treatment scheme led to improvements in the control of diurnal and nocturnal blood pressure values and OSA metrics, with reductions observed in all individuals. In cases of resistant hypertension, a 6-month CPAP treatment in patients with moderate to severe OSA resulted in a decrease of 24 h urinary aldosterone by 2.5 mg/24 h. In subgroup analysis, CPAP decreased 24 h urinary aldosterone compared to the control group, and the effect was more pronounced when spironolactone users were excluded [31].

## 4. Vascular and Hemostatic Consequences of OSA: Arterial Remodelling, Endothelial Dysfunction, and Hypercoagulability

Additionally, the increase in sympathetic tone has been associated with elevated heart rate and arterial stiffness, notably, in cases of AHI (≤15/h), higher heart rate correlates with higher brachial–ankle pulse wave velocity; conversely, within the low–heart rate subgroup, higher AHI is linked to increased brachial ankle pulse wave velocity, indicating a potential synergistic effect [32]. Increased carotid intima-media thickness (IMT) is an adaptive response that enhances arterial load and reacts to an inflammatory environment, promoting smooth muscle cell proliferation and collagen deposition. Therefore, carotid intima-media thickness, measured by ultrasound, is regarded as a non-invasive marker of subclinical atherosclerosis and endothelial dysfunction. In the ELSA cohort study, conducted in Brazil and involving 2009 subjects, the CMIT rose progressively with the severity of OSA, with a median CIMT of ~0.690 mm in subjects with no OSA (AHI < 5) compared to 0.760, 0.810, and 0.82 mm for mild, moderate, and severe OSA, respectively. In contrast, CIMT did not differ by objectively measured sleep duration (<6, 6–8, >8 h) [33].

Using CPAP for 2–24 weeks improved endothelial function, with a 3.87 percentage point increase in flow-mediated dilation based on nightly use. Conversely, stopping CPAP decreased flow-mediated dilation by 3.2%. Literature data showed that, in cases of severe OSA, CPAP has a time-dependent effect on intima-media thickness, reducing it by about 0.073 mm over a period longer than 6 months, with an average thickness of 0.121 mm [34]. CPAP improves significant artery stiffness; after 4 months of usage, carotid-femoral pulse wave velocity fell from 10.4 ± 1.0 to 9.3 ± 0.9 m/s. In symptomatic OSA, CPAP decreased the augmentation index and, with more prolonged and consistent use, also lowered pulse-wave velocity. These results suggest an improvement in arterial stiffness, although such effects were not seen in shorter or less adherent trials [35]. In an extensive cohort study with 4422 participants, respiratory events linked to cardiovascular artery disease (CAD) showed a dose-dependent increase in incident CAD among men, especially at AHI ≥ 30/h and in those aged 70 or younger. No significant link was found in women [36]. In the multicenter Sleep and Stent registry, untreated moderate to severe OSA after percutaneous coronary intervention independently predicted future major adverse cardiovascular and cerebrovascular events (MACCE, such as cardiovascular death, nonfatal myocardial infarction, nonfatal stroke, and unplanned revascularisation) over a median follow-up of 1.9 years. The 3-year MACCE rate was 18.9% for patients with OSA, compared to 14.0% for those without, with no increase in target-vessel revascularisation or stent thrombosis [37]. In revascularized patients with coronary artery disease, individuals with moderate to severe obstructive sleep apnea (OSA) who adhered to treatment for four or more hours per night exhibited an approximately 71% reduction in the adjusted risk. Event rates were approximately 2.3 versus 5.3 per 100 person-years for adherent compared to non-adherent or non-CPAP patients [38].

OSA is associated with a hypercoagulable state, which is proposed as one of the contributing mechanisms in the observed increased risk of atherosclerotic events. Data from the literature indicate that the presence of OSA is associated with an increase in fibrinogen and prothrombotic factors, accompanied by a decrease in fibrinolytic capacity [39]. Several theories have been proposed as the primary determinant of the procoagulant state, either through endothelial dysfunction or by interfering with the coagulation cascade or fibrinolysis. Intermittent hypoxia modifies hepatic protein synthesis and aggravates inflammation in the liver, the primary source of producing coagulant and anticoagulant factors [39]. Also mediated by the intermittent hypoxia episodes is the increased expression of hypoxia-inducible factor-1α (HIF-1α) and the transcription factor nuclear factor-κB, which is linked to the upregulated expression of procoagulant factors. These modifications promote thrombosis by increasing tissue factor (factor III), FVIIa/FXIIa, and fibrinogen, while also enhancing platelet activation markers, such as P-selectin and microparticles. Additionally, they raise antifibrinolytic agents such as PAI-1, α2-antiplasmin, and TAFI [39]. The concentration of factor III was directly related to the percentage of time spent with an oxygen saturation less than 90% and ODI, demonstrating the role of intermittent hypoxia in the production of the procoagulant state. Additionally, plasma levels of factor VIIa and factor XIIa were found to be significantly higher in individuals with OSA.In obstructive apnea, CPAP primarily exerts an antithrombotic effect. It consistently reduces platelet activation and, over extended therapy, lowers certain procoagulant factors. There is no change in VIIa, XIIa, or VIIIa after 1 month, but reductions in factors V, VIII, and vWF occur around 2 months. By approximately 6 months, factor VII also decreases. Global indices indicate a decrease in coagulability, as evidenced by the prolongation of PT and aPTT after approximately 30 days [39].

The effects of CPAP therapy duration and adherence appear to counteract OSA-related hypercoagulability to some extent. Short-term use, lasting around one month, is associated with reduced PAI-1 levels, decreased platelet aggregation, and slightly longer routine coagulation times; however, activated FVIIa/XIIa levels often remain stable. More prolonged use results in more extensive coagulation changes at two months, with lower levels of factor V, factor VIII, and vWF observed; however, PAI-1 levels remained unchanged [40]. The continuation of device use, after three months, exhibited a decrease in platelet aggregability, and at approximately six months, lower factor VII levels were reported. Conversely, CPAP withdrawal for two weeks leads to an increase in platelet-derived microparticles, indicating a shift back toward a prothrombotic state [40].

OSA causes a specific type of endothelial damage characterised by oxidative stress activation, eNOS uncoupling, disruption of the L-arginine and ADMA pathways, leading to reduced NO bioavailability and the initiation of inflammatory signals alongside hypoxia-adaptive responses. It also promotes a pro-thrombotic condition, results in endothelial cell apoptosis, and facilitates the release of microparticles that support the development of atherosclerosis. Furthermore, it impairs vascular repair by depleting and damaging endothelial progenitor cells [40].

In randomised studies involving individuals with mild, moderate, or severe OSA, CPAP therapy was associated with notable improvements in endothelial function. Ultrasound evaluations of flow-mediated dilation showed a 3.87% increase, while in cases of mild or minimal OSA, the improvement was 2.1% after six months [41]. Six months of adherence to positive airway pressure showed improvement in oxidative stress markers, with a soluble NOX2-derived peptide exhibiting a 24% reduction. Urinary 8-isoprostane F2-alpha, recognised as the gold standard biomarker for lipid peroxidation or oxidative stress, was found at high levels during frequent respiratory events. After therapy, his levels decreased by 23% [42].

## 5. From Nocturnal Hypoxia to Ventricular Remodelling: How OSA Drives Heart Failure

The myocardium sustains damage during recurrent episodes of hypoxia, resulting in a decline in myocardial function. During an episode of apnea, the effort made against an occluded upper airway generates significant negative intrathoracic pressure that rises left-ventricular (LV) transmural pressure while simultaneously increasing venous return. The simultaneous hypoxia-induced pulmonary vasodilatation indeed elevates right-ventricular afterload. During the diastole, the right ventricle is distended and the septum shifts toward the left, impairing left ventricular filling. The combination of higher afterload and reduced preload decreases stroke volume and cardiac output, with a blunted, delayed recovery in patients with heart failure compared with healthy individuals. In parallel, the rise in LV wall stress increases myocardial oxygen demand, while apnea-related hypoxemia and reduced coronary perfusion limit oxygen supply, fostering subendocardial ischemia, transient contractile depression, and impaired relaxation [43]. Repetition of these cycles across the night promotes adverse cardiac remodelling, hypertrophy, and progression of heart failure. Left-ventricular pressure-strain loop assessment using two-dimensional speckle tracking is an innovative technique that detects subtle subclinical changes by evaluating myocardial deformation and afterload, making it a valuable tool in various disease assessments. This analysis revealed severity-dependent subclinical left ventricular dysfunction, despite a preserved ejection fraction. In severe OSA cases, subclinical systolic impairment and less efficient myocardial mechanics were observed, even with maintained ejection fraction. In conclusion, AHI remained independently associated with lower total left ventricular systolic work and reduced constructive myocardial work, supporting an actual OSA-related decrease in effective left ventricular performance [37]. Additionally, incident heart failure in individuals diagnosed with OSA showed an adjusted incidence of 1.13 per 10-unit increase in AHI and 1.58 for AHI ≥ 30 compared to <5 in males, whereas no significant association was found in women [44]. When discussing acute heart failure, a cohort study found that 7% of subjects had a secondary diagnosis of OSA; however, in cases of heart failure with preserved ejection fraction, it was associated with longer length of stay (6.45 versus 5.79), higher odds of acute kidney injury, and atrial fibrillation [45]. Incident diastolic dysfunction (defined by E/e > 14) has been linked to OSA severity, with long-term progression towards left ventricular diastolic dysfunction driven by OSA severity and sleep fragmentation. Subjects with moderate–severe OSA (AHI ≥ 15; 26.0%) exhibited worse baseline diastolic indices (higher E/e′, higher LVMI with preserved LVEF) and a markedly higher incidence of future diastolic dysfunction compared to those with none–mild OSA (28.4% vs. 10.6%) [46]. After statistical analysis, it was confirmed that moderate to severe OSA is associated with a greater risk of diastolic dysfunction [47]. The evaluation of sleep apnea–hypoxic burden revealed that, although it was associated with AHI and a higher severity, the correlation weakened, indicating heterogeneity in hypoxic exposure that could not be explained solely by the frequency of events. Men exhibited a higher mean apnea-hypopnea index (AHI) and a higher mean apnea-hypopnea hypoxic burden. Over 10.4 ± 3.4 years, 543 participants developed heart failure; the incidence was 11.9/1000 person-years in men and 9.2/1000 person-years in women [48]. Regarding the heart failure phenotype, OSA is common in preserved ejection fraction heart failure, affecting 60% of individuals with preserved ejection fraction.

In a case–control study of heart failure with preserved ejection fraction (HFpEF), sleep-disordered breathing was found in 64% (16 of 25) of patients compared to 12% (3 of 25) of matched controls. Among those with HFpEF, OSA was the most common, present in 13 of 16 cases (81%), and AHI severity was positively correlated with diastolic dysfunction measures [49]. Similar data was found in a prospective HFpEF cohort of 244 patients, where 69.3% experienced sleep-related respiratory disturbances, with 39.8% having OSA. The severity ranged from mild (40%), moderate (36%), to severe (24%), indicating diverse levels of respiratory issues among HFpEF patients [50].

In cases of congestive heart failure with reduced ejection fraction accompanied by sleep breathing disorders, using adaptive servo ventilation for three months suppressed sleep apnoea and improved sleep quality. However, it did not change left-ventricular ejection fraction, heart failure class, or other standard heart failure indices during follow-up, nor did it affect cardiovascular outcomes [51]. In a cohort study including 55 Japanese individuals with heart failure with reduced ejection fraction and moderate to severe OSA, one month of therapy not only improved sleep disorders but also significantly enhanced left ventricular function. LVEF increased from 37.2 ± 9.8% to 43.2 ± 11.7%, averaging about 6% per month. The benefits were more pronounced in younger participants and those with higher BMI [52].

## 6. Obstructive Sleep Apnea and Arrhythmogenesis: Autonomic Imbalance, Triggers, and Substrate

OSA is associated with characteristic heart rate variability (HRV), a signature of autonomic imbalance, characterised by reduced parasympathetic tone (lower HF power, RMSSD, and SDNN) and increased sympathetic predominance (higher LF power and LF/HF ratio). These alterations in HRV are linked to the severity of OSA, being more pronounced in severe OSA, which showed significantly lower HF, RMSSD, and SDNN, and higher LF and LF/HF ratios versus controls. Moderate OSA differed primarily in having a higher LF/HF ratio. Furthermore, the HRV analysis during the day reveals nighttime elevations in LF and LF/HF, and daytime reductions in HF, RMSSD, and SDNN (with higher LF/HF) in individuals with OSA compared with controls. Taken together, these findings support a severity-dependent shift toward sympathetic dominance and vagal withdrawal in OSA [53]. CPAP can be used as a means of temporarily rebalancing cardiac autonomic control. Evidence shows that after one night of treatment by counteracting the hypoxia-driven sympathetic overactivity, results persist for 2–4 weeks in the case of more than 30 events per hour. In moderate-to-severe COPD, non-invasive ventilation using BiPAP devices can acutely shift HRV toward reduced vagal tone [54].

Arrhythmogenesis in OSA involves both an immediate trigger during each obstructive event and a long-term substrate that maintains the initial acute changes. During an apnea episode, parasympathetic dominance causes sinus bradycardia, pauses, and atrioventricular blocks, while the atrial effective refractory period shortens due to muscarinic activation. At the end of an apneic episode, the withdrawal of parasympathetic overstimulation, combined with a sympathetic surge, leads to tachycardia and a transient increase in blood pressure, which can trigger atrial or ventricular ectopy. Night-time intrathoracic pressure swings (from −10 mmHg to −15 mmHg) activate baroreflex-mediated vagal responses and cause atrial stretch, both of which further shorten the atrial effective refractory period and promote re-entry [55]. Excessive stretch activates new currents, and calcium overload favours premature contractions. Surges in afterload and hypoxemia decrease subendocardial oxygen supply, while increased wall stress raises demand, transiently depressing contractility and disrupting repolarisation. Chronic intermittent hypoxia causes electrical remodelling by shortening action potentials, slowing conduction, and delaying afterdepolarisation. Repeated cycles of hypoxia and reoxygenation lead to the activation of ROS, HIF-1α, and NF-κB, resulting in inflammation and fibrosis, which in turn cause atrial enlargement and impaired left atrial strain. Chemoreflex sensitisation induces chronic sympathetic dominance and atrial hyperactivity, amplifying both triggers and substrate. Additionally, the RASS/aldosterone loop promotes fluid retention and myocardial or atrial fibrosis, worsening OSA and increasing the risk of arrhythmias [55,56].

In individuals with severe OSA, the daytime corrected QT interval was 10.0 ms longer, and abnormal QT intervals occurred in 34% of men and 31% of women. These findings support ventricular repolarisation abnormalities as a plausible pathway linking severe OSA to increased arrhythmic and sudden cardiac death risk [57]. Individuals with moderate to severe OSA who had a catheter ablation for paroxysmal atrial fibrillation showed no difference in AF occurrence after 12 months of CPAP therapy compared to those receiving standard care only [58]. Another study involving OSA individuals with AF found that CPAP therapy was linked to a 42% relative reduction in atrial fibrillation recurrence. Similar outcomes were observed with pulmonary vein isolation and medical treatment alone [59]. In patients with heart failure with reduced ejection fraction and coexisting OSA, CPAP affects ventricular arrhythmias. A one-month CPAP treatment resulted in a 58% decrease in ventricular premature beats, accompanied by improvements in OSA severity, blood pressure, sympathetic activity, and LVEF, which increased from 27.6% to 34.3%. Similar findings were reported by Domaradzki et al. In severe OSA cases, three months of CPAP adherence resulted in a reduction of 24 h premature ventricular contractions and non-sustained ventricular tachycardia. The most significant benefits were observed in patients with nocturnal-dominant PVCS and nocturnal oxygen saturation levels below 80%. Notably, a night/day PVC ratio of ≥1.16 was an indicator of likely responders [59]. In a cohort of 15 outpatients from 15 OSA patients with severe bradycardia who adhered to CPAP therapy, a complete resolution of bradycardia and conduction abnormalities was seen in all 11 cases within 3–4 days. This suggests that bradyarrhythmias associated with OSA can be rapidly reversed with CPAP [60]. In the MOSAIC randomised trial involving adults with minimally symptomatic and mild OSA, mostly mild by ODI, six months of CPAP did not enhance resting ECG markers related to atrial or ventricular arrhythmic risk, suggesting no impact on AF risk indicators [61].

## 7. Obstructive Sleep Apnea and Pulmonary Hypertension: Mechanisms, Phenotypes, and CPAP Effects

Pulmonary hypertension (PH) is a condition characterised by a mean pulmonary artery pressure exceeding 20 mmHg at rest, as measured by right heart catheterisation. The classification of PH includes five categories: the first consists of the idiopathic form, as well as those linked with connective tissue disease, congenital heart disease, and other conditions. The association of sleep-related hypoxia in this group was common, and sleep-related hypoxia was profound. For every additional 10% of sleep time spent with less than 90% oxygen, there was an associated increase in right ventricular systolic pressure by 2.5 mmHg, a mean pulmonary artery pressure rise of 1.9 mmHg, and a drop in right ventricular ejection fraction of approximately one percentage point. These changes were consistent with 26% higher odds of right ventricular hypertrophy on ECG [62]. Additionally, sleep-related hypoxic burden independently predicted worse transplantation-free survival, whereas AHI and nadir SpO2 were not prognostic. Group 2 encompasses PH due to left heart disease, while group 3 includes PH caused by lung disease and/or hypoxia, under which OSA falls. Imran et al. discovered that in group 3 PH, over a period of 3 to 70 months of using CPAP, there was an average reduction of 13.3 mmHg in pulmonary artery pressure among 222 individuals studied. Group 4 is chronic thromboembolic pulmonary hypertension, and group 5 includes PH with unclear or multifactorial mechanisms [62]. The pathophysiological changes that occur during an apnoeic event lead to stress-triggering hypoxic pulmonary vasoconstriction and sustained sympathetic and RASS activation, raising pulmonary arterial tone. Due to chronic exposure, the pulmonary circulation undergoes vascular remodelling (proliferation of pulmonary arterial smooth muscle cells and fibroblasts, medial thickening and luminal narrowing driven by oxidative stress and inflammation, PPARγ inhibition, and an imbalance of vasoactive mediators [63]. Patients with OSA-PH overlap had a lower FEV1/FVC (%) compared with OSA individuals without pulmonary hypertension. They were also associated with male sex, older age, a higher body mass index, and a greater apnea-hypopnea index, compared with those without OSA. Among patients with OSA hospitalised for acute decompensated heart failure, pulmonary hypertension with a pulmonary artery systolic (PASP) pressure exceeding 36 mmHg was present in 61% of patients. Conversely, 18% had severe hypertension with a PASP of 60 mmHg or higher. Additionally, the presence of pulmonary hypertension was linked to a high readmission rate of 55% within the following 90 days and was associated with higher 15-month mortality [64].

## 8. Conclusions

Beyond being a comorbidity, OSA acts as a modifiable risk factor that reflects disease severity and hypoxic burden. Extensive evidence links intermittent hypoxia, sleep fragmentation, significant fluctuations in negative intrathoracic pressure, activation of the sympathetic nervous system and renin–angiotensin–aldosterone system (RAAS), endothelial dysfunction, oxidative stress, and hypercoagulability to various hypertension phenotypes—particularly non-dipping or resistant hypertension—as well as atrial and ventricular arrhythmias, progressive arterial stiffening, CIMT, incident coronary artery disease (notably in men with AHI ≥ 30/h), heart failure (with sex-specific differences), and pulmonary hypertension. Continuous positive airway pressure (CPAP) therapy reliably normalises nocturnal rebreathing. It improves intermediate vascular and autonomic parameters, such as reductions in nocturnal blood pressure (especially in non-dippers and resistant hypertension), enhancements in endothelial function, favourable shifts in heart rate variability, and decreases in prothrombotic and oxidative markers. However, large secondary prevention trials primarily involving populations with coronary artery disease (CAD) or acute coronary syndrome (ACS) who are not symptomatic have shown neutral outcomes regarding major adverse cardiac events (MACE), unless treatment adherence exceeds four hours per night. Routine cardiovascular care should incorporate targeted screening for obstructive sleep apnea (OSA), especially in cases of resistant hypertension, atrial fibrillation, and heart failure. Additionally, it should include prompt sleep testing and adherence-oriented CPAP therapy, in conjunction with conventional risk-reduction strategies.

## Appendix A

**Table A1:** Main studies included in this review, summarizing design, population, objectives, key findings, outcome domains, and publication source.

Year	Study (First Author)	Design/Population	Aim/Purpose	Key Findings (Concise)	Outcome Area	Journal
2019	Benjafield [2]	Literature-based global analysis	Estimate global OSA burden	~936 Million adults (30–69) with OSA; highest counts in USA, China, Brazil, India	Epidemiology/Prevalence	Lancet Respir Med
2011	Hermida [21]	Prospective cohort; treated hypertensives undergoing 24 h ABPM; median follow-up ≈5.6 years	To determine whether sleep-time BP reduction predicts/decreases CV risk	Each 5 mmHg decrease in sleep-time systolic BP associated with ≈17% reduction in CV events (*p* < 0.001), independent of other ABPM parameters	Cardiovascular events (composite)	J Am Coll Cardiol
2019	Sapiña-Beltrán [26]	Hypertensive OSA cohort	CPAP effect by circadian BP pattern	Greater nocturnal BP fall; partial restoration of dipping in non-dippers	Hypertension/CPAP	Eur Respir J
2021	Crinion [24]	Normotensive adults	OSA and non-dipping; reversibility	Non-dipping correlates with OSA severity; improves with therapy	Hypertension/Phenotype	ERJ Open Res
2023	Loh [29]	Systematic review/meta-analysis	RAAS activity in OSA	Higher RAAS markers in OSA; CPAP may reduce RAAS activation	Mechanistic/RAAS	J Sleep Res
2016	Krasińska [30]	Resistant HTN + OSA	Eplerenone effects	Reduced AHI, neck circumference, BP, and arterial stiffness	Hypertension/Intervention	Pol Arch Med Wewn
2014	Nicholl [31]	OSA without comorbidities	CPAP and RAAS	CPAP lowered aldosterone (men) and norepinephrine; reduced BP	Mechanistic/RAAS	AJRCCM
2017	de Souza [32]	Resistant HTN + OSA (RCT)	CPAP on aldosterone excretion	CPAP reduced 24 h urinary aldosterone	Hypertension/CPAP	J Hypertens
2021	Souza (ELSA-Brasil) [34]	Population cohort	OSA vs. CIMT	CIMT increases stepwise with OSA severity	Vascular/CIMT	ATVB
2017	Chen [35]	Meta-analysis	CPAP and CIMT	CPAP reduces CIMT over >6 months (~0.073 mm)	Vascular/CPAP	PLoS One
2015	Schwarz [40]	Systematic review/meta-analysis	CPAP and endothelial function	CPAP improves FMD by ~3–4 percentage points	Vascular/Endothelium	Respirology
2012	Del Ben [41]	OSA cohort	Oxidative stress and endothelium	PAP reduces oxidative stress; improves endothelial function	Vascular/Oxidative	BMC Pulm Med
2016	Lee (Sleep and Stent) [38]	PCI registry	OSA and MACCE post-PCI	Untreated moderate–severe OSA → higher 3-yr MACCE (18.9% vs. 14.0%)	CAD/Outcomes	Circulation
2016	Peker (RICCADSA) [65]	RCT, CAD + non-sleepy OSA	CPAP and CV outcomes	Neutral overall; adherence ≥ 4 h/night associated with benefit	CAD/CPAP	AJRCCM
2010	Gottlieb (SHHS) [37]	Community cohort	OSA and incident CHD/HF	OSA associated with incident CHD/HF in men	CAD/HF incidence	Circulation
2022	Hunt [56]	RCT post-PVI with OSA	CPAP and AF recurrence	No overall reduction vs. usual care	AF/CPAP	Heart Rhythm
2015	Shukla [57]	Meta-analysis	OSA treatment and AF recurrence	CPAP linked to ~42% lower AF recurrence	AF/CPAP	JACC Clin Electrophysiol
2020	Walker [55]	Cross-sectional	QTc in severe OSA	~10 ms longer daytime QTc; more abnormal QTc	Arrhythmia substrate	Sleep Disord
2020	Azarbarzin [46]	Prospective cohorts	Hypoxic burden and HF	Hypoxic burden predicts incident HF better than AHI	HF/Prognosis	Chest
2022	Kidawara [45]	Prospective cohort	OSA and diastolic dysfunction	OSA severity/fragmentation predict incident diastolic dysfunction	HF/Diastolic	JAHA
2020	Gupta [47]	HFpEF case–control	SDB prevalence/type	SDB 64% in HFpEF (vs. 12% controls); OSA predominant	HFpEF/Phenotype	Monaldi Arch Chest Dis
2023	Wang [48]	HF by EF class	Prevalence by EF; event type	OSA more common in HFpEF/HFmrEF; central events in HFrEF	HF/EF phenotype	Sleep Breath
2022	Tamisier (FACE) [49]	HF (mixed) + SDB	3-mo ASV effects	Better sleep metrics; no change in LVEF/HF class/outcomes	HF/Therapy	Thorax
2022	Naito [50]	HFrEF + OSA cohort	CPAP and LVEF	~6% absolute LVEF rise in 1 month (more in younger/high BMI)	HFrEF/CPAP	Front Neurol
2016	Imran [62]	Meta-analysis (isolated OSA)	CPAP and pulmonary pressures	Mean PAP reduction ~13 mmHg (3–70 mo CPAP)	PH/CPAP	Heart Fail Rev
2023	PVDOMICS [61]	Group 1 PAH	Sleep hypoxia and outcomes	Hypoxic burden predicts RV dysfunction and worse survival	PH/Prognosis	JACC
2024	Feng [18]	Cross-trait GWAS + MR	Genetic OSA liability and CVD	Genetic OSA liability associates with higher AF/CVD risk	Genetics/OSA→CVD	Eur J Prev Cardiol
2024	Gao [19]	Two-sample MR	Mediation of BMI→AF	~22% via OSA, ~49% via leptin; combined ~88% (BMI→AF to null)	Genetics/Mediation	Front Nutr
